# Atorvastatin for preventing the progression of sepsis to severe sepsis (ASEPSIS Trial): a randomised, double-blind, placebo-controlled trial (ISRCTN64637517)

**DOI:** 10.1186/cc9688

**Published:** 2011-03-11

**Authors:** JM Patel, C Snaith, D Thickett, L Linhortova, T Melody, P Hawkey, T Barnett, A Jones, T Hong, G Perkins, M Cooke, F Gao-Smith

**Affiliations:** 1 Heart of England NHS Foundation Trust, Birmingham, UK

## Introduction

Statins have pleiotrophic effects independent of their lipid-lowering properties and may modulate the pathophysiology of sepsis, prevent sepsis progression and improve outcomes [1]. This study evaluated the acute use of Atorvastatin in reducing sepsis progression compared with placebo in statin-naive individuals.

## Methods

A single-centre, randomised placebo-controlled, double-blind trial (RCT). Ethical approval and consents were obtained. Patients with sepsis, based on the Surviving Sepsis Campaign Guidelines (SSCG), were randomised to Atorvastatin 40 mg daily or placebo for length of hospital stay or 28 days if earlier. Patients on statins were excluded. Primary outcome was progression to severe sepsis, defined by the SSCG.

## Results

**Figure 1:**
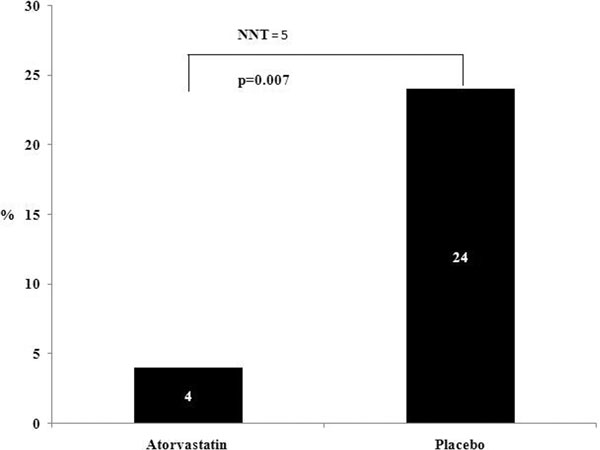
**Percentage of patients progressing to severe sepsis (%)**.

One hundred patients were consented and randomised, 49 to Atorvastatin and 51 to placebo. Both were well matched for all baseline characteristics. The Atorvastatin group had a lower rate of sepsis progression *P *= 0.007 (Figure 1). The 28-day and 1-year mortalities were similar with an overall 12% mortality. There was no difference in 28-day readmissions (*P *= 0.83); however, 1-year readmissions were higher in the placebo group (*P *< 0.001). A rise in matrix metallopeptidase 9 (*P *= 0.01) at day 4 was observed in the Atorvastatin group.

## Conclusions

This is the first RCT to show that the acute use of Atorvastatin can prevent sepsis progression in statin-naive individuals. A multicentred RCT is required to elucidate the mechanisms and clinical applications of these findings.
